# Classification of red cell dynamics with convolutional and recurrent neural networks: a sickle cell disease case study

**DOI:** 10.1038/s41598-023-27718-w

**Published:** 2023-01-13

**Authors:** Maxime Darrin, Ashwin Samudre, Maxime Sahun, Scott Atwell, Catherine Badens, Anne Charrier, Emmanuèle Helfer, Annie Viallat, Vincent Cohen-Addad, Sophie Giffard-Roisin

**Affiliations:** 1 ENS Lyon, Lyon, France; 2 Aix Marseille Univ, CNRS, CINAM, Marseille, France; 3 Aix Marseille University, INSERM, Marseille Medical Genetics (MMG), 13005 Marseille, France; 4 Sorbonne Universités, UPMC Univ Paris 06, CNRS, LIP6, Paris, France; 5 Univ. Grenoble Alpes, Univ. Savoie Mont Blanc, CNRS, IRD, IFSTTAR, ISTerre, Grenoble, France

**Keywords:** Cellular imaging, Diagnostic markers, Sickle cell disease, Computer science, Biological physics

## Abstract

The fraction of red blood cells adopting a specific motion under low shear flow is a promising inexpensive marker for monitoring the clinical status of patients with sickle cell disease. Its high-throughput measurement relies on the video analysis of thousands of cell motions for each blood sample to eliminate a large majority of unreliable samples (out of focus or overlapping cells) and discriminate between tank-treading and flipping motion, characterizing highly and poorly deformable cells respectively. Moreover, these videos are of different durations (from 6 to more than 100 frames). We present a two-stage end-to-end machine learning pipeline able to automatically classify cell motions in videos with a high class imbalance. By extending, comparing, and combining two state-of-the-art methods, a convolutional neural network (CNN) model and a recurrent CNN, we are able to automatically discard 97% of the unreliable cell sequences (first stage) and classify highly and poorly deformable red cell sequences with 97% accuracy and an F1-score of 0.94 (second stage). Dataset and codes are publicly released for the community.

## Introduction

The high deformability of red blood cells (RBC) is a key factor for proper blood microcirculation and is often impaired in hemolytic anemia. In particular, the physiopathology of Sickle Cell Disease (SCD), a group of highly handicapping inherited RBC disorders that affects more than 4.5 million people worldwide^1^, is associated with, among other mechanisms, a decrease in RBC deformability. The latter plays a major role in the clinical outcome, particularly in the occurrence of typical painful and unpredictable vaso-occlusive crises leading to infarction and tissue ischemia that can be life-threatening^2^. The molecular cause is a variant of the hemoglobin molecule, which, under deoxygenation and dehydration self-assembles into fibers in the RBC cytoplasm thus increasing its viscosity^3^. This increase leads to a decrease in RBC deformability that contributes to their blockage in the microcirculation. The self-assembling process is only partially reversible over time, thus the RBC of an SCD patient have very heterogeneous cytoplasmic viscosities and deformability, although most cells keep an overall normal biconcave shape^4^. This last point shows the limits of a method that would analyze the shape of RBC to determine their deformability. Up to now, several methods addressing RBC deformability have been described to monitor the variations of this RBC feature in SCD patient blood samples: for instance, microfluidic devices with constrictions can be used to discriminate normal RBC shapes from sickle ones, based on their transit or recovery time^5–8^; also the Oxygenscan (also called oxygen gradient ektacytometry) assesses RBC deformability as a function of a continuous variation of di-oxygen pressure^9^. All these techniques require either intensive-labor handling of individual cells, or the need to use sophisticated or delicate devices that cannot be easily used in patients’ homes^10^. Thus, a simple and rapid method sensitive to the different parameters governing RBC deformability and valid for heterogeneous RBC populations is still lacking. Yet, a close monitoring of red cell deformability may help to prevent complications and improve patient health.

**Figure 1:**
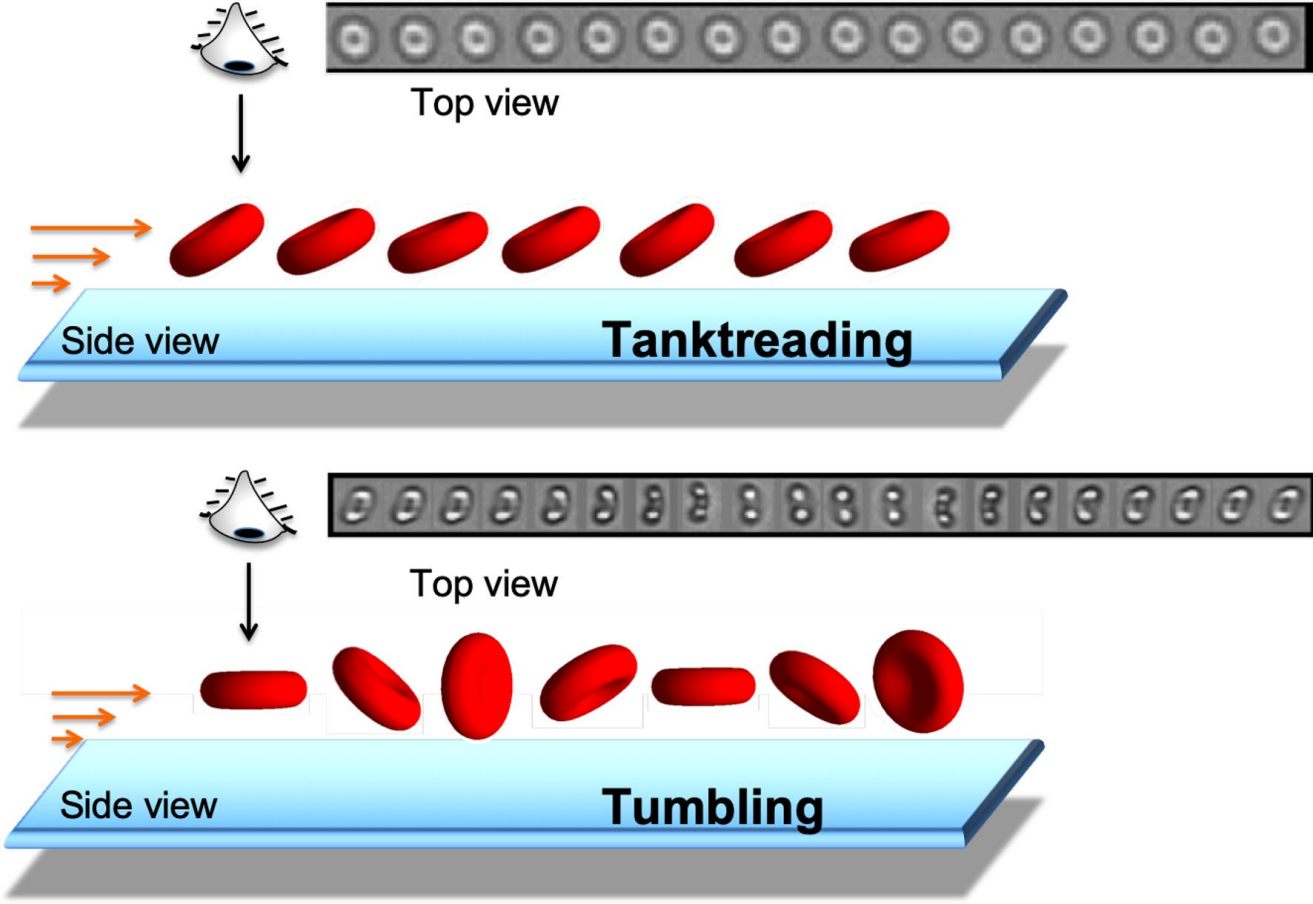
RBC motions under shear flow (orange arrows) near a wall. Top: tank-treading motion of a highly deformable cell. The cell keeps a constant orientation with respect to the flow direction (direction of the orange arrows). The cell membrane rolls like the caterpillar of a tank; schematic representation: projection of the cell in the shear plane, ‘side view’, and example of a time sequence of 16 images of a RBC obtained by videomicroscopy: projection of the tank-treading cell perpendicular to the shear gradient direction, top view. Bottom: non tank-treading motion, here flipping, of a poorly deformable RBC. The cell tumbles in the flow like a coin, its orientation changes with respect to the flow direction; schematic representation: projection of the cell in side view and example of a time sequence of 20 images of a RBC obtained by videomicroscopy: projection of the tumbling cell in top view. Image acquisition frequency: 60 images/sec.

In this study, we exploit the fact that the individual movement of an RBC in shear flow is an indicator of its deformability^11,12^. Under an increasing shear rate, the motion of RBC suspended in a high viscosity medium (typically 40 mPa.s) changes from a rigid-like flip-flopping motion (like a tumbling coin) to a wheel-like rolling motion before transiting above a critical shear stress to a stationary droplet-like tank-treading motion (see figure 1)^11,13–15^. During tank-treading, the membrane of the RBC rotates around its center of mass and its orientation oscillates around a mean value. In conjunction with these experiments, models and numerical simulations showed that the critical shear stress of the transition from rolling to tank-treading directly depends on a combination of membrane shear-elasticity, membrane and cytoplasm viscosities and RBC aspect ratio^16–20^. The higher the RBC deformability, the lower the critical shear stress to reach tank-treading. In a recent study, it was shown that for the same density, RBC from SCD patients, even with a normal biconcave shape, need a higher critical shear stress to reach tank-treading motion than those from healthy patients, a behavior that can be inferred to deformability reduction due to the increase of internal viscosity resulting from hemoglobin polymerization in SCD cells^21^. Another recent study used a simple macroscopic flow chamber coupled with videomicroscopy to show that, at a given shear stress, 0.39 Pa, the fraction of tank-treading RBCs of a blood sample suspended in a 39-mPa.s viscosity medium strongly differs between control and SCD patients^22,23^. Moreover, the study demonstrated that this parameter is stable over six months in patients outside periods of vaso-occlusive crises and significantly varies during crises, with a net decrease 2-3 days before the crisis. The simplicity of the device and method, which do not require micron-scale flows, paves the way for a low-cost, easy-to-implement bedside system to monitor the clinical status and predict vaso-occlusive crises of patients with SCD^23^. More detailed information on the results and the method can be found in the “Patent description” section.

However, such a test relies on analyzing the individual motion of thousands of RBC for each patient, extracted from a videomicroscopy recording of a large population of RBC circulating through the field of view of the camera. The analysis must discriminate between tank-treading motion (highly deformable RBC), flipping motion (poorly deformable RBC), and a large majority of unreliable samples (out of focus cells or cells overlapping during the recording, or other experimental artifacts). Moreover, the individual cell motion videos are of different durations (from 6 to more than 100 frames) which increases the complexity of the classification.

The need of automatic cell classification in biology has led to an important number of image processing techniques. First, classical vision methods (such as thresholding, edge detection, mathematical morphology) have shown to work in some static image cases^24^. Nevertheless, in the last decade, image and video classifications have been revolutionized by machine learning methods, able, for example, to classify images between thousands of categories^25^ or videos between more than 400 categories^26^. They both rely on convolutional neural networks, a deep learning technique that is suited for spatially coherent data. It has also been shown that such methods can be very powerful when dealing with human cell imaging tasks. For example, Gao et al.^27^ and Phan et al.^28^ classify images of staining patterns of Human Epithelial-2 (HEp-2) cells, while Chen et al.^29^ distinguishes white blood T-cells against colon cancer cells. Liang et al.^30^ developed a deep learning model able to classify four types of blood cell images (lymphocyte, monocyte, neutrophil, and eosinophil). More importantly, Xu et al.^31^ developed a method to classify the different RBC shapes of sickle cell disease patients observed at rest. It does not provide information about alterations in the deformability of ‘normal’ biconcave-shaped red cells that nevertheless have degraded deformability.

However, there is a clear lack of techniques for cell *motion* classification from video input data (2D+t). While there is a large body of work on cell tracking in time-lapse biology for various applications such as mitosis or apoptosis detection (see e.g.:^32,33^ and the recent survey of^34^), and some work on distinguishing *croissants*-shaped from *slippers*-shaped red blood cells in Poiseuille flows^35^, none of them appear applicable to our setting where the goal is to identify the *motion* (tank-treading or not) of the cell in very short videos sequences (as opposed to the shape). Other related applications consists of cell tracking and segmentation from time image series: He et al.^36^ developed an iterative convolutional neural network (CNN) to track living deformable cells on videos with frames every 5 minutes. Su et al.^37^ proposed an interesting automatic method for detecting mitosis events in microscopy videos. They used a convolutional long-short-term memory (LSTM) network that couples CNNs with LSTMs, a type of recurrent deep learning method capable of storing memory particularly suited for temporal series data. But to our knowledge, no study has attempted to classify different cell motions, in particular in the context where the video sequences have varying number of frames.

In this work, we propose an end-to-end two-step machine learning pipeline able to automatically classify the cell motion videos, even with different time lengths and with a high imbalance between the classes. By extending, comparing, and combining two state-of-the-art methods, namely a CNN model and a recurrent CNN, we are able to automatically discard 97% of the unreliable cell sequences (first step) and classify highly deformable vs. poorly deformable blood cell sequences with a 97% accuracy and F1-score of 0.94 (second step).

The article is structured as follows. Sec. “Methods” presents the data, the pre-processing steps, the two steps task and the proposed machine learning models. Finally, Sec. “Results” details the qualitative and quantitative results of both stages.

## Methods

All methods were carried out in accordance with relevant guidelines and regulations. All experimental protocols were approved by the institutional review board Comité de Protection des Personnes Ouest 6 under the reference n$^{\circ }$2018A00679-46. Informed consent was obtained from all subjects and/or their legal guardian(s).

### Clinical data description

**Blood samples.** Four adult patients with SCD were used in the study. They were enrolled in the study Drepaforme and were sampled weekly for several months. Two patients had genotype hemoglobin SS, and two patients had genotype hemoglobin S$\beta$0. Blood was collected by finger prick (3 $\mu$L), directly diluted in 1 ml of dextran solution (see below) and homogenized by gentle stirring. The suspension was used within the day. Just before use, it was diluted by a factor of 25 in the same dextran solution as before.

**Dextran solution.** Dextran (from Leuconostoc mesenteroides, 2000 kDa, Sigma-Aldrich) was solubilized at 9% (wt/wt) in DPBS+glucose (40 mM) at an osmolarity of 295 ± 5 mOsm (controlled by adding glucose) and pH = 7.4, by stirring at 50 °C for at least 2 h. The Dextran solution had a viscosity equal to 39.2 ± 0.7 10$^{-3}$ Pa.s at 20 °C and its density approximately matched that of RBC, thus preventing cell sedimentation.

**Flow experiments and microscopy.** Flow experiments were performed as described previously^14^. Briefly, the blood suspension was injected in a parallelepiped quartz flow chamber (50$\times$10$\times$1 mm$^{3}$, Hellma, France) mounted on an inverted microscope (DMIRB, Leica). The fluid was driven by a syringe pump (11 Plus, Harvard Apparatus) at a controlled flow rate to set a wall shear rate equal to 10 s$^{-1}$. RBC were observed in bright-field microscopy (10$\times$ objective with additional $\times$1.5 magnification) 2 cm away from the chamber entrance, at 60 $\mu$m from the bottom wall (zone of constant shear rate) along the direction of the flow gradient. The videos of RBC crossing the camera field (830$\times$655 $\mu$m$^{2}$) were recorded directly on the computer at 60 frames per second and an exposure time of 2.2 ms with a camera (Infinity 3-6UR, QImaging). As shown in sequences in Figure 1, each video image displays the projection of the cell along the direction of the flow gradient. When the cell tanktreads, it keeps a fixed orientation and its projection remains the same over time. When the cell flips, its orientation with the flow direction periodically varies with time, resulting in temporal changes in the shape of its projection. All experiments were performed at room temperature (21 ± 2 °C) under oxygenated conditions. More details on flow of the suspension can be found in the “Data generation method” section.

### Trajectories extraction

The video images were recorded as 24-bit AVI files and then saved as TIFF files. The movies were processed using in-house routines in Matlab (Matlab, R2016a). RBC were detected individually and tracked over time. Briefly, a background image not containing any moving objects was created by calculating the median image of 100 random frames equally distributed in the movie. The background was then removed from every frame. Objects were detected in each frame after thresholding the image on which a variance filter was applied. RBC tracks were formed using a strong assumption on possible trajectories: objects may only move from left to right (acquisition convention) with a linear motion along the X-axis with a steady speed. Precise positioning was achieved through the cross-correlation of sequential frames. The dilution of the blood sample was chosen as a compromise between having a large number of RBC in the camera’s field of view and allowing the RBC to be sufficiently apart so that individual trajectories were detected with limited neighbor interference issues. The temporal sequence of images of each cell as it passed through the camera field was recorded in an individual file. Sequences with less than six images were discarded because they indicated a problem (interference between cells, cell not detected during its entire crossing of the camera field). Then the images of each selected sequence were automatically normalized to 31x31 pixels. These sequences were hand-classified and used as a training dataset for machine learning. For each video, between 250 and 2000 RBC were detected. However, many RBC were not in the focal plane of the microscope objective and thus appeared blurry. Cells that were not sufficiently sharp, i.e., for which we could not determine their motion regime with certainty, had to be discarded.

### Sequences preprocessing

The trajectory sequences are of various lengths ranging from 6 to more than 100 frames, mainly depending on the speed of the blood cell in the device. Since the information to extract from a sequence is always the same, down-sampling the larger sequences can reduce memory consumption and increase the classification efficiency while keeping nearly the same amount of information. In order to bound the maximum length of sequences, we developed two different downsampling methods. We consider this upper bound *K* as a hyperparameter that will be tested in the experiments (*K* varies from 10 to 50). This range was chosen because the mean sequence length is less than 40, and the few sequences larger than 70 frames only correspond to the unreliable class (to be discarded).

**Uniform downsampling.** It consists in uniformly removing images from the sequence in order to get the desired number of images *K*.

**Similarity downsampling.** It consists in keeping the most dissimilar images of the sequence. Indeed, for human expertise, the discrimination between tank-treading and flipping RBC is done on the temporal evolution of the shape of the cell projection during the sequence (Figure 1). Therefore the most relevant information of the sequence comes from the dissimilarity between images. We are thus interested in removing consecutive images that are too similar. To do so, we used the structural similarity index to assess the similarity between two consecutive images. We then kept the ones with the lowest similarity with an iterative process.

**Short sequences.** For the sequences having a number of images smaller than K, the processing differs depending on the methodology applied (CNN or recurrent CNN, see below): for the CNN method, the input size is fixed so the short sequences will be padded to get exactly K images (see section “Approach A: Fixed-size convolutional neural networks). For the recurrent CNN, the input sequences can be of different sizes so no modification is performed.

### Two-stage deep learning classification

**Figure 2:**
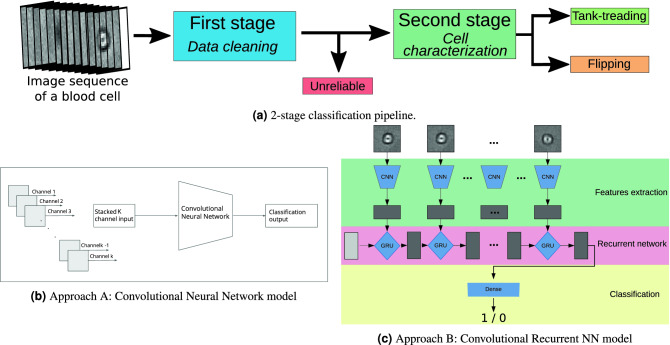
The overall 2-stage classification pipeline (**a**) and the two deep learning approaches developed and compared for both first and second stages (**b**,**c**).

The sequences are cell-centered trajectories. However, in many cases, they can be partially corrupted because of artifacts in some images such as blurred cells, partial representation of the cell, or overlapping of multiple cells into a single image. Unfortunately, it seems extremely challenging to automatically correct these sequences and much easier to simply discard these unreliable sequences from the rest of the data since the remaining ones still allow for an accurate final prediction. We thus have now three classes: the *unreliable* sequences, the sequences of tank-treading RBC, hereafter called *tank-treading* sequences, and the sequence of flipping RBC, hereafter called *flipping* sequences. Because of the highly imbalance classes (the *unreliable* being largely over-represented) and because the classification between *reliable* and *unreliable* is an easier task, we split our problem in two stages, see Fig. 2. Both stages are 2-class classification techniques: the first one aims at cleaning the data (*reliable* vs. *unreliable*, section “First stage: data cleaning”), and the second aims at characterizing the cells (*poorly deformable* vs. *highly deformable*, section “Second stage: Cell characterization”). We developed, trained and compared deep learning classification techniques on both stages, and we selected the hyperparameters separately for each stage.

### First stage: data cleaning

In the first stage of the classifier, we aim to separate the sequences labeled as unreliable from the others: the dataset is divided into two categories, the *unreliable* and the *reliable* (consisting of both the *flipping* and *tank-treading* sample sequences).

We observe a large imbalance (15 to 30 times more *unreliable* than *reliable*) between the number of samples in the two classes. This imbalance can perturb the training process, as it leads to a model that classifies well the dominant class and ignores the other since they are negligible. We will see in the implementation details section “Implementation details” the different methods to overcome this issue.

### Second stage: Cell characterization

In the second stage, we aim to classify the reliable sequences into *tank-treading* and *flipping*. It is the actual disease detection phase. For training the models to perform this task, we extracted, by human expertise, the sequences with ground truth as either *tank-treading* or *flipping* from our dataset. There are 5 to 15 times more *tank-treading* than *flipping* sequences.

### Machine learning approaches

The input data is composed of sequences of grayscale images, and an appropriate machine learning model is needed to handle these two characteristics (*image* and *temporal sequence*, a.k.a. 2D+t). Convolutional Neural Networks (CNN) have shown good results in blood cell classification from images^28–30^. However, very few studies dealt with 2D+t blood cell images; and none studied the classification of cell motions. To deal with the sequential characteristic, we propose two approaches. Our first method (approach A) consists of a standard CNN where the stacked channels are used to embed the time dimension. Our second method (approach B) uses Recurrent Neural Networks (RNN) to handle sequences of varying sizes and aggregate information over the whole sequence, as RNNs have achieved interesting results in sequence processing^38^.

We trained and tested both approaches (A and B) on both stages (1 and 2) to design an end-to-end sequence processing method that removes unreliable sequences (Sec. “First stage: data cleaning”) and distinguishes between sequences labeled *tank-treading* featuring highly deformable RBC and the ones labeled *flipping* featuring poorly deformable RBC (Sec. “Second stage: Cell characterization”). First, we describe those 2 model architectures, and then in Sec. ‘Protocols and experiments’ we present their results.

### Approach A: Fixed-size convolutional neural networks

After resizing the sequences to the fixed number *K* of frames, we train a CNN model with the number of input channels equal to *K*. Usually, CNNs are used on images with multiple color channels (such as Red, Green, Blue) whose shapes are (N_channels, height, width). Since we are dealing with grayscale images (with only one channel), we propose to use the channel dimension to embed the temporal dimension of the data: the first channel is used for the first image of the sequence and so on (see Figure 2).

**Padding Sequences Lengths.** The preprocessed sequences are equal or shorter than *K*. To get only sequences of size exactly *K*, we pad sequences of length smaller than *K* with black images placed at the end of the sequence to obtain sequences of length exactly *K*.

**CNN architecture.** We used the ResNet-18 architecture^39^, which improved the standard CNN models by using some ‘skip connections’, solving the vanishing gradient problem in deep architectures. The choice in the architecture was made by testing different state-of-the-art architectures (9-layer CNN, VGG-16, ResNet-18, ResNet-34, ResNet-50) and selecting the best performing model. It is a sequence of 20 layers alternating convolutional layers, pooling layers, a fully connected layer, and a softmax layer at the end (see Table 1 left). The convolutional layers extract spatial features by convolving the input image with different spatial filters or kernels. The pooling layer reduces the spatial size of the representation, while the fully connected layer is a dense layer where each output neuron is connected to every input neuron. All hidden layers are equipped with the rectification (ReLU) non-linearity activation. The four convolutional blocks (ConvBlock) contain two sets of 3x3 kernel convolutions, while the first convolution is of size 7x7.

**Table 1:** Architectures of the 2 CNN models. *s* stands for *stride*: step of the convolution over the input. *p* stands for *padding*: addition of zeros to avoid shrinking of the input during convolution operation. Left: Approach A, the CNN architecture ResNet. Right: Approach B, Architecture of the feature extractor (see Figure 2).

Layers	Type	Parameters
1	Convolutional layer	64 kernels (7*x*7), s = 2, p = 3
	Activation	Relu
1	Max Pooling layer	Size (3,3)
2-5	ConvBlock1	[64x(3x3)] x 2, s = 2, p = 1
	Activation	Relu
6-9	ConvBlock2	[128x(3x3] x 2, s = 2, p = 1
	Activation	Relu
10-13	ConvBlock3	[256x(3x3)] x 2, s = 2, p = 1
	Activation	Relu
14-17	ConvBlock4	[512x(3x3)] x 2, s = 2, p = 1
	Activation	Relu
18	Average pooling layer	Size (1*x*1)
19	Fully connected layer	1000x(output feature map)
20	Softmax activation	Output probabilities
1	Convolutional layer	8 kernels (3*x*3), s = 1, p = 1
2	Activation	Relu
3	Convolutional layer	32 kernels (2*x*2), s = 1, p = 1
4	Activation	Relu
5	Convolutional layer	64 kernels (2*x*2), s = 1, p = 1
6	Activation	Relu
7	Max Pooling layer	Size (3*x*3)
8	Convolutional layer	128 kernels (3*x*3), s = 1, p = 1
9	Activation	Relu

### Approach B: Convolutional Recurrent Neural Networks

Approach A does not use a specific architecture to deal with the sequential characteristics of the data. It can lead to two main downsides. As stated previously, it requires sequences of the same length; moreover, it may lose the temporal information since it processes all the images of the sequence together. On the contrary, approach B uses a recurrent neural network (RNN) where connections between nodes form a directed graph along a temporal sequence: it allows to exhibit temporal dynamic behavior.

Concretely, approach B is a combined version of CNN and RNN, namely a convolutional recurrent network (C-RNN). First, we transform the sequence of images into a sequence of 1D feature maps extracted by a CNN (Figure 2) applied independently on each frame. This step outputs a sequence of feature vectors summarizing each image. Then, we build an RNN (using gated recurrent unit cells^40^) to aggregate the temporal information of a sequence in a meaningful temporal way. It leads to a representation of the sequence in a single vector, embedding the relevant information of each image. Eventually, we feed this vector into a dense neural network which finally classifies the sequence (Figure 2). While this method consists of different steps, the training is performed simultaneously.

Note that this architecture enables us to process sequences of arbitrary size, eliminating the need to pad the shorter sequences.

**Feature extractor.** We used the CNN architecture presented in Table 1 right, simpler than the ResNet of Approach A since the input is here a single image. While we are representing in Figure 2 different CNN blocks because the feature extraction is performed separately, the model is unique and the model weights are shared between the different frames.

**Recurrent Model.** Then the vectors are fed into a *Gated Recurrent Unit* (*GRU*) network with 64 units, producing a unique *context vector* of size 64. In order to aggregate the information contained in the sequence of features vectors, we used GRU cells^40^. This state-of-the-art RNN can extract information from significantly larger sequences than standard recurrent networks and are faster than *Long Short Term memory* cells networks^41,42^. While processing a given sequence, a GRU unit maintains a context vector updated at each step by removing redundant or unnecessary information and extracting relevant information from the input. This context vector forms a relevant summary of the sequence until the current step. GRU units then produce an output based on this summary of the past elements of the sequence and the current one.

**Final Classification.** Lastly, the *context vector* is passed to a dense neural network with two layers of 64 and 2 neurons outputting a binary vector: the final class prediction.

### Implementation details

**Artificial class balance.** In both stages, the classes are highly imbalanced. As a large data imbalance can perturb the training of deep learning models, we tested different methods to artificially balance the training data. The first method is to downsample the majority class in order to keep only a subset of the class randomly. The second method consists in oversampling the minority class by copying the existing samples. After empirically testing both methods, we downsampled the majority class for approach A. In approach B, the best performance was found by upsampling the minority class. Moreover, for stage 1 in approach B, a combination of both methods gave the best performance (downsampling from 70 000 to 5 000 the *unreliable* cells and upsampling from 3 800 to 5 000 the *reliable* cells). This artificial balance was only performed on the training set, and the evaluation was done on the *real* setting, i.e. imbalanced configuration.

**Training loss.** We trained our models by minimizing the categorical cross-entropy loss^43^ between the predicted classes and the true classes. Let $p(\cdot | x)$ be the probability distribution over the classes $\fancyscript{y}$ output by our model for input *x* and $q(\cdot | x)$, the true distribution for *x*, *i.e.*, a Dirac distribution representing *x* true class. Then, given an input set $\fancyscript{D}$, the mean categorical cross-entropy is defined as

1 $$\begin{aligned} {\text {CrossEntropy}}(p, q) = \frac{1}{|\fancyscript{D}|} \sum _{x\in \fancyscript{D}} \sum _{y \in \fancyscript{y}} p(y|x) \log (q(y|x)). \end{aligned}$$

We trained our models with a fixed number of epochs (i.e. iterations). After each epoch, we evaluated the model on the validation set. The epoch reaching the best loss on the validation set is then selected as the best model for that run.

**Model hyperparameters.** We used Fastai^44^ library, PyTorch^45^ and Nvidia P100 graphics processing unit (GPU) for the hardware support. We used Adam optimizer for weight updates during training. The learning rate was selected using the cyclical learning rates^46^ method and thus set differently between the range of $1e^{-4}$ to $1e^{-3}$ for various layers in the network. This helps in the faster training of the model. The batch size was set to 64 and the number of epochs was between 20 and 40, based on the values that performed best on the validation set.

**Computation time.** In Approach A, the running time for training varies based on the sequence size from 30 minutes (for $K=10$, 1.5 minutes per epoch) to 1.75 hours (for $K=50$, 5 minutes per epoch). In Approach B, in contrast, the training time is less than 30 min for 40 epochs (less than a minute per epoch), moreover, this model converges in nearly 20 epochs, improvements after that are minimal.

### Evaluation setting

#### Dataset splitting

We have processed the sequence data for four patients organized in different dates of blood collection. There is a total of 32 experiments (acquisitions). We separated the experiments and selected 19 for training, 4 for validation, and 9 for testing. All 4 acquisitions from test Patient 1 are kept unseen by the training and validation phases. Table 2 summarizes the number of sequences of each label in the three sets. The training set was used to train the models, the validation set was used to select the best hyperparameters (Section “Model selection and hyperparameters tuning”), and the test set, kept unseen, was used to give the final evaluation results (Section “Results analysis”).

**Table 2:** Dataset splitting.

Label	Training set	Validation set	Testing set
tank-treading (reliable)	3229	649	1714
flipping (reliable)	550	95	125
unreliable	69071	18251	52815

#### Model selection and hyperparameters tuning

**Hyperparameters.** The two stages (data cleaning Sec. “First stage: data cleaning” and cell characterization Sec. “Second stage: Cell characterization”) are different and thus could require different models and hyperparameters. For both stages, we tested the two model approaches, as well as different sequence preprocessing (uniform or similarity downsampling) and different sequence size (*K*). We recall that for the C-RNN method, *K* is only the maximal sequence size: for sequences smaller than *K*, the size remains untouched. We used a common validation set (See Sec “Protocols and experiments”) to evaluate these configurations and select the best for each stage.

**Metrics.** To assess the efficiency of each configuration, we evaluated different classification metrics. The accuracy, defined as the ratio of the number of correctly predicted sequences to the total number of sequences, is not sufficient because of the imbalance of the classes for both stages. For example in stage 1, the *unreliable* sequences are over-represented: a model predicting every sample as *unreliable* would still reach a $95\%$ accuracy. In order to avoid such behaviours, we used the F1-score to select the best model, and we calculated the precision and recall^47^ as well as the confusion matrices for the final evaluation. Precision is the fraction of relevant instances (here *reliable* for stage 1 and *flipping* for stage 2) among the retrieved instances whereas recall, also called sensitivity, is the fraction of relevant instances that were retrieved. The F1-score is the harmonic mean of precision and recall:

2 $$\begin{aligned} F_1 = 2 \times \frac{{\text {precision}} \times {\text {recall}}}{{\text {precision}} + {\text {recall}}} \end{aligned}$$

### Model selection for stage 1

Table 3 summarizes the F1-score, accuracy, precision and recall on the validation set for the different models for the data cleaning stage. We can see that both approaches (A and B) have similar performances, with high accuracy and recall, but smaller precision. This is due to the fact that the dataset is highly imbalanced (from Table 2: 744 *reliable* with respect to 18251 *unreliable* validation samples), see section “Results analysis” for the analysis of the final results. We can see that for Stage 1, K is more influential than the sampling type. In terms of model selection, the C-RNN with K=50 gives the best F1-score and precision, while the CNN with K=20 has the best accuracy and recall. Since F1-score is a good balance between precision and recall, we selected for stage 1 the model B: C-RNN with K=50 and uniform sampling.

**Table 3:** Stage 1: F1-score, accuracy, precision and recall on the validation set for different models, K values and sampling methods (uni = uniform; sim = similarity). In bold, the best value for each metric. Top-left: F1-score. Top-right: Accuracy. Bottom-left: Precision. Bottom-right: Recall.

		A: CNN		B: C-RNN	
		*sampling*		*sampling*	
		uni.	sim.	uni.	sim.
*K*	10	0.48	0.47	0.56	0.55
	20	0.54	0.55	0.56	0.56
	30	0.50	0.51	0.58	0.58
	50	0.52	0.53	**0.61**	**0.61**
*K*	10	0.92	0.89	0.95	0.95
	20	**0.99**	**0.99**	0.95	0.95
	30	0.93	0.94	0.95	0.95
	50	0.94	0.94	0.96	0.96
*K*	10	0.32	0.30	0.39	0.38
	20	0.36	0.38	0.40	0.39
	30	0.31	0.33	0.42	0.42
	50	0.33	0.35	**0.46**	**0.46**
*K*	10	0.93	0.90	0.97	0.96
	20	0.97	**0.98**	0.96	0.95
	30	0.92	0.94	0.95	0.95
	50	0.94	0.95	0.94	0.93

### Model selection for stage 2

Similarly, we performed a hyperparameter search to select the best model for the cell characterization stage based on the validation set results. We recall that here, the relevant class for calculating the precision and the recall is the *flipping* class (the minority group). From Table 4, we can see that the best F1-score is achieved using model A (CNN), with K=20 and similarity sampling: this setting will be selected. It is interesting to notice that the two stages need different settings to achieve a good performance. For this second stage, large values (K=50) of maximal sequence size are not satisfactory, giving low precision results for model A and low recall results for model B.

**Table 4:** Stage 2: F1-score, accuracy, precision and recall on the validation set for different models, K values and sampling methods (uni = uniform; sim = similarity). In bold, the best value for each metric. Top-left: F1-score. Top-right: Accuracy. Bottom-left: Precision. Bottom-right: Recall.

		A: CNN		B: C-RNN	
		*sampling*		*sampling*	
		uni.	sim.	uni.	sim.
*K*	10	0.80	0.81	0.91	0.89
	20	0.92	**0.95**	0.93	0.90
	30	0.82	0.82	0.93	0.89
	50	0.77	0.80	0.78	0.87
*K*	10	0.83	0.85	**0.98**	0.97
	20	0.94	**0.98**	**0.98**	**0.98**
	30	0.85	0.87	**0.98**	0.97
	50	0.79	0.81	0.96	0.97
*K*	10	0.79	0.80	0.90	0.90
	20	0.91	0.93	0.92	0.91
	30	0.82	0.80	0.96	0.91
	50	0.75	0.78	**1.00**	0.97
*K*	10	0.82	0.84	0.92	0.88
	20	0.93	**0.96**	0.94	0.89
	30	0.83	0.85	0.90	0.87
	50	0.79	0.81	0.68	0.79

## Results

### Stage 1: data cleaning results

#### Quantitative results

**Figure 3:**
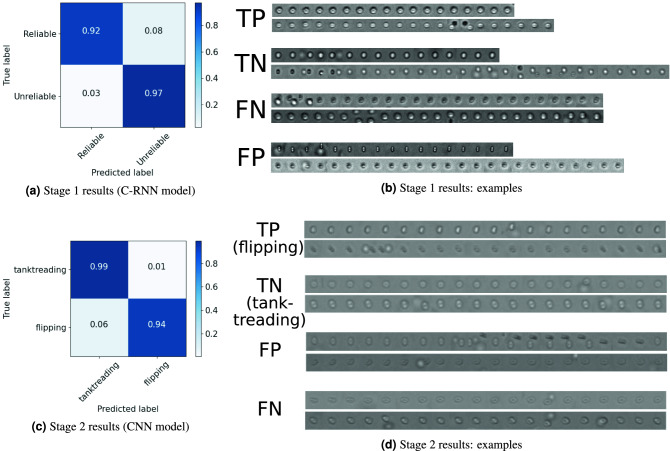
(Right) Normalized confusion matrix (Left) 8 sequence examples of true positive samples (TP), true negative samples (TN), false negative samples (FN) and false positive samples (FP). (**a**) This representation indicates that 92% of the *reliable* samples were correctly predicted (8% of false negatives) and 97% of the *unreliable* samples were correctly predicted (3% of false positives). The test set is composed of 1839 *reliable* and 52815 *unreliable* samples. (**b**) TP: *reliable* samples correctly classified as *reliable*; TN : *unreliable* samples correctly classified as *unreliable*; FN: *reliable* samples incorrectly classified as *unreliable*; FP: *unreliable* samples incorrectly classified as *reliable*. (**c**) This representation indicates that 99% of the *tank-treading* samples were correctly predicted (1% of false positives) and 94% of the *flipping* samples were correctly predicted (6% of false negatives). The test set is composed of 1714 *tank-treading* and 125 *flipping* cell sequence samples. (**d**) TP: *flipping* samples correctly classified as *flipping*; TN : *tank-treading* samples correctly classified as *tank-treading*; FP: *tank-treading* samples incorrectly classified as *flipping*; FN: *flipping* samples incorrectly classified as *tank-treading*. Here we represent the sequences after the similarity sampling (K=20) for simplicity.

The C-RNN model selected in section “Model selection and hyperparameters tuning” was applied on the unseen testing set and the results are summarized in the confusion matrix of Figure 3 (top left). The F1-score is 0.67, the accuracy 0.97, the precision 0.52, and the recall 0.92. As can also be seen in the confusion matrix, the high recall indicates that almost all *reliable* are correctly classified: the data cleaning does not miss many *reliable* samples, which is of major importance. The precision reaches however only 0.52, indicating that 48% of the C-RNN detections are in fact *unreliable*. This is because the number of *unreliable* is nearly 30 times superior than the number of *reliable* samples. While a final manual data cleaning would still be needed, our automated data cleaning thus removes 97% of the *unreliable* samples.

#### Qualitative results

By analyzing the classification errors of the data cleaning model in Figure 3 (top right), we can see that the false negatives (FN, *reliable* samples that were incorrectly classified as *unreliable*) are often partially corrupted sequences that are similar to some *unreliable* sequences (such as the true negatives, TN). The decision boundary between *reliable* and *unreliable* samples is tenuous, and performing a validation from different experts would show how precise we can expect an algorithm to be.

### Stage 2: cell characterization results

#### Quantitative results

Similarly, the CNN model selected in Section “Model selection and hyperparameters tuning” was applied on the unseen testing set (without the ground truth *unreliable* samples) and the confusion matrix is shown in Figure 3 (bottom left). The F1-score is 0.94, the accuracy 0.97, the precision 0.93, and the recall 0.94. While this stage also deals with class imbalance, the CNN model shows both good precision and recall. This is because the imbalance is smaller, but also because the *flipping* and *tank-treading* classes might be more separable. Lastly, we estimate for the test set the percentage of *highly deformable cells* vs. the total number of cells, which is the final measure needed to estimate the state of a subject. The CNN predictions give a final *highly deformable cells* percentage of 93.5%, while the ground truth percentage is 93.2% : we can conclude that the automatic characterization gives a precise estimation of the sickle cell disease measure.

#### Qualitative results

Figure 3 (bottom right) shows correctly classified and misclassified examples of the cell characterization. We can see that the CNN model can separate *flipping* from *tank-treading* sequences even where there is noise in some frames (for example when there is a second cell out-of-focus). Nevertheless, we can see in the misclassified examples that too much noise can lead to failure, such as in the first example of the false positives (FP): the secondary cell that appears from the middle of the sequence is probably perceived by the network as the ‘flipping’ of the first cell, leading to label the sequence *flipping* instead of *tank-treading*. In terms of false negatives (FN), it seems that when the ‘flipping’ happens very early in the sequence, such as in the first FN example, it can be missed by the CNN model.

#### SCD marker

fraction of *tank-treading* cells. The potential inexpensive test to monitor the clinical condition of SCD patients is the fraction of *tank-treading* cells that are *tank-treading* in a patient acquisition. In the test set, the cell sequence samples are taken from 9 acquisitions from two different patients. We thus estimated for every acquisition the percentage of *highly deformable cells* classified by the stage 2 deep learning model vs. the total number of *reliable* cells, and compared this number to the ground truth ratio, see Table  5. We can see that the percentage error is mostly below mostly below 2%, the largest error (3.3%) being found on the smallest acquisition set (120 cell sequences).

**Table 5:** Percentage of *highly deformable cells* vs. the total number of *reliable* cells for the 9 acquisitions of the test set. Ground truth ratio and estimated ratio (classified by the stage 2 deep learning model) are compared. *# samples* is the total number of *reliable* cell sequences of each acquisition.

	Test patient 1				Test patient 2				
	*acquisitions*				*acquisitions*				
	1	2	3	4	1	2	3	4	5
Ground truth	92.9	94.8	94.7	92.4	95.2	93.3	86.4	95.4	92.7
ratio (%)									
Estimated	91.4	93.9	93.6	91	95.2	90	84.2	94.4	90.4
ratio (%)									
# samples	255	229	266	212	146	120	177	216	218

## Discussion

Our results provide some interesting insights regarding the optimal sequence downsampling technique with respect to the task. While uniform downsampling performs well for the data cleaning (stage 1), it performs poorly for the cell characterization (stage 2). This might be because in stage 2, the algorithm aims to detect the cell movement so the dissimilar moving frames should absolutely be kept by the downsampling method. We think that in other applications, such analysis can be important in order to select an appropriate measure. Moreover, the best architecture differs also for the two tasks. This is probably since for the first task, a large maximal sequence size K can help in detecting the amount of *noise* in the sequence which is needed to discard the *unreliable* samples: large sequences are easily processed in a recurrent network than in a standard CNN. Recently, some more complex models, such as Transformer networks, based on the attention mechanism, have shown interesting results in sequence processing. A future perspective will be to test such methods on this dataset. As we have seen, the data cleaning is the more challenging task, and in future work, we also seek to perform experiments with smaller numbers of *unreliable* sequences, because it can directly impact the estimation of the final ratio of *highly deformable* vs. *poorly deformable* cells. The dataset is publicly available here 10.5281/zenodo.5723606; and our neural network pipeline is also publicly released along with pretrained models and the code to test the pipeline in the form of Jupyter notebooks at github.com/icannos/redbloodcells_disease_classification.

We showed that deep learning models were able to accurately classify blood cell sequences in a two stage pipeline, a data cleaning followed by a cell movement characterization, from sequences of various lengths and with very unbalanced datasets. We showed that the dynamics within a sequence can be learned from two different deep learning architecture families: CNN and C-RNN. Both models were relevant for one of the stages, showing the importance of model selection for every task. Last, we showed that on the test set our model gives a value of the tank-treading RBC fraction (93.5%) that is very similar to the ground truth tank-treading RBC fraction (93.2%): this metric might thus be automatically estimated in order to monitor the clinical condition of SCD patients. However, the high number of unreliable sequences can be a limitation. The improvement of the sequence acquisition could significantly lower this number and give a better confidence on the final SCD maker, and will thus be investigated in future works. We believe that these results could open the way to other research applications dealing with biological dynamics classification.

## Patent description

The following paragraph summarizes the results of the patent Viallat et al., 2022^23^, which has been thoroughly externally reviewed. It is shown on 9 controls and 14 sickle cell patients that almost all RBC from controls (mean values of 99%) adopted a tank-tread motion while only a fraction of RBC from sickle cell patients did (mean value 0.70 ± 0.12) with no overlapping distributions. It is also shown on several patients that this ratio measured at the time of hospitalization during a vaso-occlusive crisis was significantly lower than the range observed out of a crisis obtained from a 6-month weekly observation of this ratio, i.e. mean value ± twice the standard deviation. A “master” curve was also obtained, indicating a net decrease of this ratio 2-3 days before the crisis. The marker is sufficiently reliable to be used in the context of sickle cell disease. We showed that this marker is patient-specific.

## Data generation method

The RBC concentration of the suspension was chosen low enough that the cells do not interact with their neighbors located in the same horizontal plane. However, the velocity of a cell depends on its distance from the bottom wall so that distant ones can catch up and overtake the ones closer to the bottom wall. When a fast cell overtakes a slow one, a temporary interaction is possible but this only concerns a few images and we have never observed any destabilization of motion. These few images are eliminated during the tracking and are not taken into account for the determination of the motion. The number of these images is small enough to never completely mask a flip of a cell that could induce an error on its type of movement.

## Accession codes

The dataset is publicly available here 10.5281/zenodo.5723606; and the neural network pipeline is also publicly released along with pretrained models and the code to test the pipeline in the form of Jupyter notebooks at github.com/icannos/redbloodcells_disease_classification.
